# On the space of SARS-CoV-2 genetic sequence variants

**DOI:** 10.18699/VJGB-23-97

**Published:** 2023-12

**Authors:** A.Yu. Palyanov, N.V. Palyanova

**Affiliations:** A.P. Ershov Institute of Informatics Systems of the Siberian Branch of the Russian Academy of Sciences, Novosibirsk, Russia Research Institute of Virology, Federal Research Center of Fundamental and Translational Medicine of the Siberian Branch of the Russian Academy of Sciences, Novosibirsk, Russia Novosibirsk State University, Novosibirsk, Russia; Research Institute of Virology, Federal Research Center of Fundamental and Translational Medicine of the Siberian Branch of the Russian Academy of Sciences, Novosibirsk, Russia

**Keywords:** coronavirus, SARS-CoV-2, genome, space of variants, evolution, variability, коронавирус, SARS-CoV-2, геном, пространство вариантов, эволюция, изменчивость

## Abstract

The coronavirus pandemic caused by the SARS-CoV-2 virus, which humanity resisted using the latest advances in science, left behind, among other things, extensive genetic data. Every day since the end of 2019, samples of the virus genomes have been collected around the world, which makes it possible to trace its evolution in detail from its emergence to the present. The accumulated statistics of testing results showed that the number of confirmed cases of SARS-CoV-2 infection was at least 767.5 million (9.5 % of the current world population, excluding asymptomatic people), and the number of sequenced virus genomes is more than 15.7 million (which is over 2 % of the total number of infected people). These new data potentially contain information about the mechanisms of the variability and spread of the virus, its interaction with the human immune system, the main parameters characterizing the mechanisms of the development of a pandemic, and much more. In this article, we analyze the space of possible variants of SARS-CoV-2 genetic sequences both from a mathematical point of view and taking into account the biological limitations inherent in this system, known both from general biological knowledge and from the consideration of the characteristics of this particular virus. We have developed software capable of loading and analyzing SARS-CoV-2 nucleotide sequences in FASTA format, determining the 5’ and 3’ UTR positions, the number and location of unidentified nucleotides (“N”), performing alignment with the reference sequence by calling the program designed for this, determining mutations, deletions and insertions, as well as calculating various characteristics of virus genomes with a given time step (days, weeks, months, etc.). The data obtained indicate that, despite the apparent mathematical diversity of possible options for changing the virus over time, the corridor of the evolutionary trajectory that the coronavirus has passed through seems to be quite narrow. Thus it can be assumed that it is determined to some extent, which allows us to hope for a possibility of modeling the evolution of the coronavirus.

## Abstract (ru)

Пандемия коронавирусной инфекции, вызванная вирусом SARS-CoV-2, которой человечество противостояло с использованием новейших достижений науки, оставила после себя в том числе обширные генетические данные. Ежедневно начиная с конца 2019 г. в мире собирались образцы геномов вируса, что предоставляет возможность детально проследить его эволюцию с момента возникновения до настоящего времени. Накопленная статистика результатов экспресс-тестирования показала, что число подтвержденных случаев заражения SARS-CoV-2 составило не менее 767.5 млн (9.5 % нынешнего населения Земли без учета бессимптомников), а число секвенированных геномов вируса – более 15.7 млн (что составляет чуть более 2 % от общего числа заразившихся). Эти новые данные потенциально несут в себе информацию о механизмах изменчивости и распространения вируса, его взаимодействия с иммунной системой человека, об основных параметрах, характеризующих механизмы развития пандемии, и многое другое. В этой статье мы анализируем пространство возможных вариантов генетических последовательностей SARS-CoV-2 как с математической точки зрения, так и с учетом биологических ограничений, присущих этой системе (основанных на общебиологических знаниях и учитывающих особенности данного конкретного вируса). Для этого мы разработали программное обеспечение, способное загружать и анализировать нуклеотидные последовательности SARSCoV- 2 в формате FASTA, определять позиции 5’ и 3’ UTR, число и расположение неидентифицированных нуклеотидов (“N”), осуществлять выравнивание относительно референсной последовательности посредством вызова предназначенных для этого программ, определять мутации, делеции и вставки, а также рассчитывать различные характеристики геномов вирусов с заданным шагом по времени (дни, недели, месяцы и т. д.). Полученные данные свидетельствуют о том, что, несмотря на кажущееся математическое многообразие возможных вариантов изменения вируса во времени, коридор эволюционной траектории, которым прошел коронавирус, представляется достаточно узким. Это дает основание полагать, что он в некоторой степени детерминирован, что позволяет надеяться на возможность моделирования эволюции коронавируса.

## Introduction

The possibility of computational modeling of the evolution, life cycle and reproduction of the simplest biological organism down to the gene level would be a scientific breakthrough, but it is still far beyond the capabilities of modern supercomputers. The process of natural selection of the fittest individuals takes into account a huge number of factors in both the external and internal environment. The characteristics of an organism are realized through sets of protein characteristics and features, and the impact of changes in each protein on an organism’s fitness is quite difficult to assess due to the need to take into account all the resulting changes in the interactions of a protein with all environmental factors and other proteins, the number of which is very significant.

Usually, in computer models of evolving objects, changes to the genome of descendants are not carried out directly (by reproducing molecular mechanisms), but are only simulated by describing algorithms for making changes to a copy of the genome of ancestors. However, the mechanisms of introducing mutations and horizontal gene transfer themselves are subjects of evolution, and among possible changes that do not lead to the death or sterility of an individual, there are also those that affect the speed and accuracy of genome replication. Due to this, intraspecific competition arises, as a result of which, for example, in the case of SARS-CoV-2, from the moment of its appearance to the present time, the duration of the incubation period, directly related to the rate of virus replication, is constantly decreasing (Malone et al., 2022).

In comparison with cellular life forms, viruses are substantially simpler and thus are much more convenient for investigation and computational modeling of their evolution, especially taking into account their significantly smaller genomes and, at the same time, still quite wide range of interactions with the external environment and host organism. Before the appearance of fast genome sequencing technologies, evolution of viruses could only be considered within the framework of “parasite–host” models, which described statistical, but not molecular features of their interaction. Since the beginning of the SARS-CoV-2 pandemic, the number of confirmed cases of this infection has been at least 767.5 million (9.5 % of the current world population, excluding asymptomatic people) (Palyanova et al., 2022). During this period, the global scientific community has obtained more than 15.7 million variants of the genomes of this coronavirus (including the date of sampling and the geographical location of the place where it was collected), providing unprecedentedly extensive data on its evolution, in such quantities that were not available for any other virus before.

Based on these data, the dynamics of spread and change of the virus can be calculated not only in physical space and time, but also in the multidimensional space of possible viable variants of viral genomes with a metric determined by the minimum number of single changes (mutation, deletion or insertion) required to transform one genome into another (known as the “Levenshtein distance” or “edit distance”). The virus changes over time, including the response to vaccination and the formation of immunity in people who have recovered from the disease. This means that both the genome of the virus and its “phenotypic” manifestations change when interacting with the carrier’s body, i. e. two parallel processes occur simultaneously – both a change (spread) of a set (cloud) of points representing the virus population (at one time or another) in the space of possible RNA sequences, and a change in the very landscape of this multidimensional surface of the “fitness function” of the virus. Each point in the space of possible states corresponds to a specific nucleotide sequence, more or less different from the original reference genome (from which it all began at the end of 2019 (Wu et al., 2020)) by a certain number of changes – mutations, deletions and insertions.

Transitions can and should exist between pairs of points (in the space of viral RNA sequence variants), each of which corresponds to a viable sequence, if one of them has resulted from the other via changes that have occurred within the virus from the moment it enters the host’s body until the appearance of the next generation of virions (usually many more than one cycle of replication of the virus genome takes place before this). Most of the possible changes that occur during replication (each copy of the viral sequence has its own set) will lead to the appearance of a non-viable variant (especially deletions or insertions the length of which is not a multiple of three – i. e. those that will lead to a reading frame shift during translation). However, some changes can leave the fitness of the virus at the same level or even increase it – for example, by raising the rate of synthesis of new viral particles or increasing their number per time unit (which will give them advantage over other variants located in the body at the same time, i. e. intraspecific competition arises). The fitness function of some viral sequence can be thought of as the number of its copies existing in the human population at a given time (with or without normalization to the total number of virus copies).

Thus, the landscape of the “surface” of the (multidimensional) fitness function is formed, which may have more or less extensive “valleys” corresponding to many similar sequences (appearing as a result of small changes in the variant that first fell into this valley), surrounded by “mountains”. There are “mountain ridges” or “plateaus” (all points of which correspond to non-viable sequences) delimiting “valleys” of viable sequences and “passes” between them. Regions of nonviable sequences correspond to the cases when, for example, a virus cannot make copies of itself due to damage to the gene encoding RNA-dependent RNA polymerase (RdRp), which performs viral RNA replication, or when changes in the structure of the capsid proteins prevents the virus from forming a protein shell, as well as for many other diverse reasons. Also, presumably, there are “valleys” for which none of the sequence variants belonging to them have yet been realized, but which can be reached in the future – for example, due to the emergence of a viable recombinant strain resulting from a combination of the genomes of two not very similar variants of the virus. It is possible that this is how the initial WT strain of SARS-CoV-2 arose.

There are currently two major databases providing online access to SARS-CoV-2 genetic sequences. The largest of them is GISAID (https://gisaid.org) – Global Initiative on Sharing All Influenza Data (started in 2006) (Khare et al., 2021). Since the emergence of SARS-CoV-2 at the end of 2019, it has also become a repository for the accumulation of sequenced variants of this virus obtained by laboratories around the world. In July 2023, there are more than 15.7 million SARS-CoV-2 sequences stored in it. Another database, NCBI SARS-CoV-2 Data Hub (Sayers et al., 2022) (https:// www.ncbi.nlm.nih.gov/labs/virus/vssi/#/virus?VirusLineage_ ss=taxid:2697049), contains more than 7.7 million SARSCoV- 2 genome sequences. Such unprecedentedly vast and detailed data have never been available to humanity before, so it is necessary to extract as much useful information and knowledge as possible from their comprehensive analysis. In this work we consider only the first steps on this path, and much remains to be done.

The Nextstrain/Nextclade project (https://clades.nextstrain. org) (Aksamentov et al., 2021), which provides online tools for analysis and visualization of genetic data on various viruses, including SARS-CoV-2, is also of great importance for the scientific community of viral genome researchers. Nextclade’s functionality stands out by providing a graphical representation of the genome map of the loaded sequences, showing mutations, deletions, insertions, unidentified nucleotides (“N”) and a number of other features of each sequence, including, for example, detection of reassortant (recombinant) variants.

The description of the space of variants of SARS-CoV-2 genetic sequences fundamentally includes (a) those that we can already observe and study thanks to extensive sequencing, (b) variants from the real space of variants that have already been implemented, but have not come to the attention of researchers, and (c) other possible variants that could be realized in the future and are of particular interest, since they are potentially dangerous to humanity and it would be good to be prepared in advance for their possible appearance (rapid tests for their detection, vaccines, etc.).

Let’s now consider the most important characteristics of SARS-CoV-2 as a system, the basis of which is self-replication in the host cells, and which may be important in the future when creating its evolutionary simulator. They include the speed of genome replication (600–700 nt/s, the highest among the known speeds of viral RNA polymerases) (Shannon et al., 2020), the time of viral RNA replication ( 3 · 104 nt 600 nt/s = 50 s), the entire virus reproduction time (7–24 hours) (Grebennikov et al., 2021) and the frequency of replication errors occurrence (1.3 · 10−6 ± 0.2 · 10−6 per position, per cycle of cell infection, i. e. from the entry of the virus into a cell until the release of new virions out of it) (Amicone et al., 2022). The rate of its evolution is estimated as 8.9 · 10−4 changes per position per year (Sonnleitner, 2022), which could lead to an average of 93 changes in 3.5 years. This correlates quite well with the fact that one of the variants most distant from the reference sequence (belonging to the “Omicron” variant, obtained on June 20, 2023) has 103 substitutions (the maximum number of mutations among the variants, see the Table). The “Alpha” and the “Beta” variants differ from the reference sequence by more than 30 point mutations and more than 17 deletions. The variants that arose later have more differences. It is also noticeable that during the evolution of the virus the number of deletions increases, reaching 59 in one of the recent branches of “Omicron”.

**Table 1.**
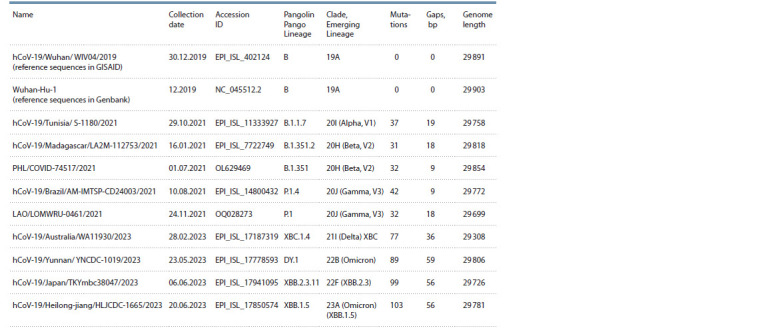
The most recent representatives of various branches of the phylogenetic tree of coronavirus SARS-CoV-2 (https://nextstrain.org/ncov/open/global/all-time) Note. Representatives of some branches (mainly belonging to different variants of “Omicron”) are still being found in sequenced specimens of SARS-CoV-2 genomes from recently infected people, and some have ceased to be detected at all (“Alpha”, “Beta”, “Gamma”, “Delta”, etc.). The reference sequences in both databases differ only in the length of the poly-A region located at the very end, and in all other positions they are completely identical.

As was already mentioned, the SARS-CoV-2 coronavirus has the fastest RNA polymerase, but it also has one of the lowest (for RNA viruses) rates of mutation occurrence during the replication process, which is necessary due to its rather large genome. This is achieved thanks to the errorcorrecting exonuclease (nsp14-ExoN), which is found only in viruses with large genomes (coronaviruses and toroviruses) (Campagnola et al., 2022).

Also among important parameters are the minimum infectious dose (the number of virions required for infection), which is about 100 particles (Karimzadeh et al., 2021), the reproductive number (1.8–3.2) (Xu et al., 2021), the number of viral particles carried by a patient during the peak of infection ((1–100) · 109) and the number of virions contained on average in an infected cell (105 ) (Sender et al., 2021), as well as other epidemiological characteristics. Viral particles are found in many tissues and organs, from the lungs to the brain, but only those present in the respiratory tract or intestines will be released and can be transmitted to subsequent carriers. All other virions will not leave “descendants”, which significantly narrows the evolutionary corridor. The works of (Day et al., 2020) and (Markov et al., 2023) addressed a number of important issues regarding the epidemiology and evolution of the SARS-CoV-2 virus, including the mechanism of the emergence of recombinant strains.

## Materials and methods

The most rational way to obtain both fast data processing speed and unlimited capabilities (which can be expanded if necessary) for their analysis, in our opinion, is to work with source FASTA files using the software package that combines our own software with third party libraries and programs. To date, a prototype that includes the minimum required functionality has been implemented. For the development, we used the C++ programming language available in Microsoft Visual Studio Community 2019. The hardware used was a PC based on an Intel Core i7-10700K processor, 3.8 GHz, 8 cores, 16 GB of RAM.

The methods used in this work mainly belong to the following two categories: – theoretical estimates and numerical calculations of some important characteristics of the system under consideration, including the quality and reliability of the data; – analysis of available genetic data using our own and existing software tools.

Whole genome genetic sequences of SARS-CoV-2. The GISAID and the Genbank databases provide, through a web interface, some functionality for studying the properties of the sequences they contain, but they are not flexible enough to perform the analysis required for investigation of the space of variants of SARS-CoV-2 genetic sequences, which is the goal of this work. There is also an API (Application Programming Interface) for GISAID (Wirth, Duchene, 2022), implemented in the R language. However, its capabilities also have limitations (including speed of operation with significant volumes of processed data) compared to direct access to genetic sequences stored as FASTA-files on a local workstation. GISAID significantly limits the possibili- ties of downloading from its website: no more than 2000 sequences per download, which completely eliminates the possibility of downloading a significant amount of data “manually”. The NCBI SARS-CoV-2 Data Hub has no such restrictions.

To analyze the already realized genetic variants of SARS-CoV-2, full-genome sequences from the GISAID (https://gisaid.org/) (Khare at al., 2021) and NCBI Virus SARS-CoV-2 Data Hub (https://www.ncbi.nlm.nih.gov/ labs/virus/) (Sayers et al., 2022) were used. Sequences from Genbank (2019–2020) were downloaded to a local workstation and analyzed using our own software developed for this purpose, named ParSeq. Because of the limitations, sequences from GISAID were not downloaded – instead we accessed them through API to obtain only some of their properties (for example, full lengths of sequences; however, we were unable to obtain viral RNAs translatable part length and the positions of its start and end).

To calculate the edit distance between pairs of SARS-CoV-2 sequence variants (including a separate calculation of the number of mutations, deletions and insertions), the Nextstrain web resource (https://clades.nextstrain.org) was used.

## Results

The estimation of the number of realized and potentially possible genetic variants of SARS-CoV-2 sequences

Let’s start with considering the space of genetic sequences from a mathematical point of view, in the most general case. Any pair of sequences can be characterized by a measure of the difference between them, called the Levenshtein distance, or edit distance – the minimum number of point (single) substitutions (mutations, deletions, insertions) that must be made in the first sequence in order to transform it into the second one. Each element of the set of sequences of a given length L has a distance between itself and the empty sequence (Ø) which is exactly equal to L. The number of variants of nucleotide sequences of length L equals 4L. The number of possible single mutations in a sequence of length L equals 3∙L (the nucleotide at each position can be replaced by any of the other three). Also, 3∙L different single deletions and 3∙(L+1) different single insertions are possible. All possible single deletions for all possible sequences of length L compose the set of all possible sequences of length (L–1), with the number of variants equal to 4(L–1). And all possible single insertions for all possible sequences of length L result in a set of all possible sequences of length (L+1), with the number of variants equal to 4(L+1).

**Fig. 1.**
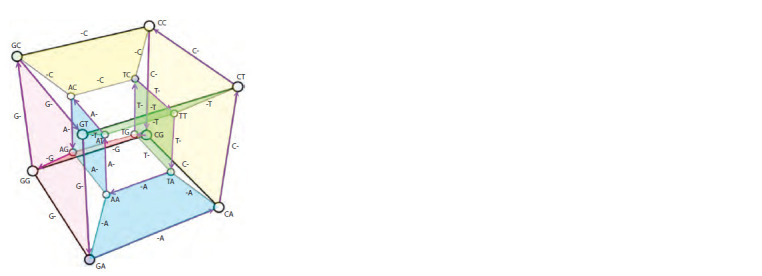
The space of nucleotide sequence variants of length = 2, represented as a hypercube One of many Hamiltonian cycles on a hypercube (purple arrows) is presented – a closed path passing through each vertex exactly once. Each transition corresponds to a single change (mutation, deletion or insertion). There are also hyperplanes that can be associated with subsequences appearing from sequence of L = 2 after single deletions from the left (-A,-T,-G,-C) or from the right (A-,T-,G-,C-), which turn out to be the same in this simple case.

**Fig. 2.**
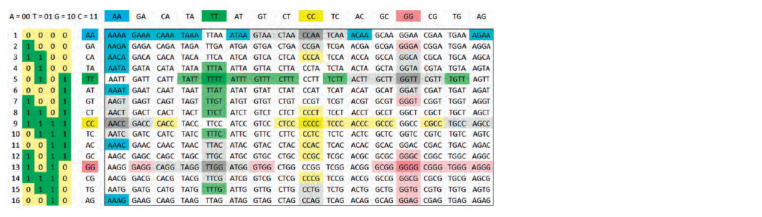
The set of nucleotide sequence variants of length 4, depicted on a plane using 2D Gray codes. The top edge of the table is coupled with the bottom, the left – with the right, i. e. one can map this set onto the surface of a torus. Then, when moving both horizontally and vertically (in the coordinate system of the table), in accordance with the properties of Gray codes, each pair of adjacent sequences will differ by exactly one (single) replacement (mutation).

Let’s consider all possible variants of nucleotide sequences of length L = 2 (Fig. 1). The set of sequences of L = 2 is quite small, but even in this simple case a hypercube in 4D space (tesseract, with 16 vertices) is required to represent all of this set’s elements. For a more complex case, L = 4, in a similar way, a 6-dimensional hypercube (hexeract) with 64 vertices can be used (however, its visualization, together with the signatures of nodes and edges, will be oversaturated with details and difficult to perceive). Nevertheless, it can be displayed, in some degree, on a 2D plane using one of the Gray codes variants (Mütze, 2023) (this theory is closely connected with hypercubes), in this case – 2D code which we were able to find for this demonstration (Fig. 2).

The usual metric, such as the sum of squared differences of Cartesian coordinates, is apparently not suitable in this case.

The number of all possible sequences of equal length, in this case – the length of the reference genome of SARS-CoV-2, L = 29903, is very huge: 429903, or approximately 2.511∙1018003. In this space of variants, the set of sequences corresponding to the realized variants of the SARS-CoV-2 genome constitutes only a small part, composed of the point corresponding to the reference sequence and its small neighborhood, currently limited by the distance from the reference sequence to the most recent “Omicron” strain. It is possible to estimate the number of possible sequence variants within this distance. For the reference sequence, with L = 29903, the number of its variations with only one single mutation = 3∙L, with two mutations = (3∙L)2 – 3∙L = 3∙L∙(3∙L–1) (from all possible cases we subtract those in which second mutation occurs in the same position as the first one, and we get one of the already existing sequences – the reference one or a sequence which differs from it in only one position). Similarly, for the third mutation: (3∙L)3 – ((3∙L)2 – 3∙L), and so on. For L = 29903, the number of all variants of sequences with a number of mutations from 0 to n (relative to the reference sequence) is equal to 1.387∙10510 for n = 103, and for L = 29847 (56 deletions) – 1.108∙10510. Summing over all lengths from 29903 to 29847, we obtain 7.190∙10511

Sequences with synonymous single nucleotide mutations that do not result in an amino acid change are also part of the total sequence variants space. However, the actual number of variants in the context of considering the structure and functions of proteins translated from viral RNA is significantly smaller due to the degeneracy of the genetic code (20 amino acids are encoded by 61 RNA triplets, i. e., on average, 3.05 triplets encode the same amino acid). Let’s also take into account that not the entire genome of SARS-CoV-2 encodes proteins: 771 out of 29903 nucleotides are non-coding. As a result, the dependence proportional to (3L)n is transformed into ≈ ((L–771)+(3·771))n and thus the corrected number of protein sequence variants can be estimated as 1.02∙10465. If we assume that someday the number of mutations will exceed the above-mentioned 103 pcs. by 10–11 times, then the sequence will most likely still be a coronavirus, but will already belong to a different species. For example, the bat coronavirus RaTG13, the closest neighbor of SARS-CoV-2 in the space of genetic sequence variants, differs from it by 1135 point mutations.

Let’s try to look at the many variants of SARS-CoV-2 genetic sequences “tested” by nature from a biological point of view. The virus gets into a body (usually by airborne droplets, ending up in the lungs) and enters a cell, where a host ribosome begins to synthesize viral proteins in accordance with the nucleotide sequence of the SARS-CoV-2 genome. Among these proteins, there is a viral RNA polymerase (RdRp), which initiates a process of viral RNA replication. At the beginning, when there is only one viral RNA and one RdRp in the cell, the probability of their meeting is extremely low, but then, as these and other molecules accumulate in the cell, it starts to grow rapidly. As a result, the concentration reaches a level sufficient for the assembly of new virions, and when their number in the cell reaches approximately 105 pieces, these virions leave it and begin to infect neighboring cells, and more distant cells as well, if some of the virions enter the bloodstream and are distributed throughout the body. Considering that the number of viral particles in a patient’s organism during the peak of infection can reach up to 1011 pcs. (Sender et al., 2021), let’s divide this value by the average number of virions in an infected cell and get the number of infected cells in the body, 106. A human being has approximately 3·1013 cells, so it appears that the percentage of infected among all is less than 10−4 %.

The frequency of errors occurrence during SARS-CoV-2 genome replication, according to (Amicone et al., 2022), is 1.3·10−6 ± 0.2·10−6 changes per position, per cell infection cycle, and is (1–2) ·10−6 according to (Markov et al., 2023), that is, approximately 1.4·10−6 on average. Taking into account the length of the sequence, we obtain the probability of a single mutation occurring in the entire sequence per replication cycle ≈ 0.04. Thus, even if all infected cells in the body contain the same viral RNA variant at some moment, then after one replication cycle the body may contain all possible variants of single substitutions (3·29903 pcs.) related to source viral RNA (which existed before the start of the cycle). So, there will be about 4 % of these (and most of them will not be viable), and 96 % will be exact copies of the replicated sequence. What will be the probability of occurrence of a viable non-synonymous mutation (changing not only the RNA sequence of the virus, but also the amino acid sequence of one of its proteins), which is also superior to its predecessor in fitness? This question remains open; however, the required probability will definitely be very small. In the vast majority of cases, all copies of the virus spread by the infected person into the external environment are identical, and only rarely two variants occur simultaneously in one organism. How then new mutant variants not only appear, but also quickly displace their predecessors on a planetary scale every now and again?

Considering that the ratio of 4 % : 96 % with each subsequent replication cycle will change towards a decrease in the proportion of mutant sequences (“founder effect” (Ruan et al., 2020)) until they completely dissappear, we can suppose the following possible scenarios (with low probabilities) for the emergence and spread of mutant variants of SARSCoV- 2:

(a) The body does not have immunity to SARS-CoV-2 since it has not yet encountered it. A single copy of the viral RNA enters the cell; during the first round of replication, a mutation arises in it, and it turns out to be viable (this indeed can happen – with a low, but non-zero probability). Then all new virions synthesized by this cell will be carriers of this mutation, and if it is noticeably advantageous, they may have a chance of displacing the initial variant.

(b) The body already has immunity against SARS-CoV-2. It simultaneously contains two variants of SARS-CoV-2 virions – the one which is dominant in the population and the new one, mutant (arising by the mechanism from (a) or a recombinant). The immune system destroys the “old” variant that is familiar to it, but the new one goes unnoticed, passes through replication cycles and is transmitted further.

The probabilities of the occurrence of these two options have yet to be estimated, but even without this it is clear that the corridor of possible variants along which evolution took place turned out to be quite narrow. The opposite of this picture is, for example, the influenza virus, the distinctive feature and basis of survival of which is high variability due to the mechanisms of antigenic drift and antigenic shift (Kim et al., 2018).

We evaluate the modeling of the evolution of SARS-CoV-2 as possible, because despite the large number of variants that should have already been realized and which could have been realized from the point of view of mathematics (probability theory) and biology, in reality only a small part of them was realized and one can observe only a small part of the possible space of variants.

The development of the ParSeq software

To analyze the genetic sequences of SARS-CoV-2, we developed the software called ParSeq (Parser of Sequences) – parser and analyzer of SARS-CoV-2 nucleotide sequences in FASTA format, which we already used while working on analysis of the SARS-CoV-2 epidemic in regions of Siberia (Palyanova et al., 2023). Its main abilities already implemented at the moment are described below:

• Loading and parsing one or many FASTA files (using the list of file names) for further analysis, including the following data fields: full-genome nucleotide sequence, “Accession ID”, “Length”, “Pango lineage”, “Nuc. completeness”, “Collection date”, “Geo location” and “Country”.

• Primary analysis of the nucleotide sequence, including calculation of its length and nucleotide content (A, U(T), G, C and non-identified nucleotides represented by the letter “N”). Also, in some sequences, the following letters of the extended alphabet are found sometimes: (https://www.bioinformatics.org/sms/iupac.html):

**Formula. 1.**
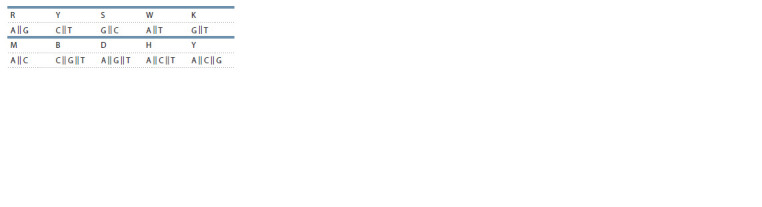
Formula 1

Determination of the positions of the beginning and end of the coding part of the sequence. In the case of a reference sequence, its total length is 29903 nt, the length of noncoding 5′ UTR – 265 nt, non-coding 3′ UTR – 229 nt. To do this, the following simple algorithm is used: in the case of a 5′ UTR, we move along the sequence from its beginning to the 500th nucleotide (for convenience, a “round” value was chosen, for which 265 is approximately in the middle) with a window of length 17 and count the number of nucleotide matches in this window with a fragment of the reference sequence of the same length, corresponding to the interval 266–282 (where 266 is the position of the translation start in the reference genome). If 14 or more out of 17 positions match, then the position is determined correctly (numerical parameters are defined as sufficient for correct operation in the vast majority of cases using a small window length – to avoid unnecessary calculations). In the case of a 3′ UTR, everything is similar – with a 17 nt long window we move along the last 500 nucleotides of the analyzed sequence, comparing its contents with the 17 nucleotides that end the coding region of the reference sequence. The criterion of the correct position is the same – 14 or more matches within the 17 nt long window.

• Calculation of the lengths of the non-coding 5′ UTR and 3′ UTR, as well as the coding region located between them, which makes up the vast majority of the genome of the viral sequence (98.35 % of its length in the case of the reference sequence).

• Calculation of distributions of these values for any selection of SARS-CoV-2 genome sequences (e. g., within a specified time interval for the collection date, or for sequences containing no more than a specified number of “N”s, etc.; combinations of various filters are also supported).

• Calculation of distribution of sequences by number of their lengths.

The results obtained using ParSeq software

Using the software we developed, we analyzed the nucleotide sequences of SARS-CoV-2, available to users around the world thanks to the Genbank and GISAID projects. As a result, the following facts were established.

1. The calculation of the distribution of genetic sequences by their full lengths (5′ UTR + coding sequence + 3′ UTR) among sequences with a length ≥ 28000 revealed that for data from Genbank (for the period from 01.12.2019 to 31.12.2022) the minimum length of the complete sequence was 28784, and the maximum was 29985. The vast majority of the distribution corresponds to lengths less than or equal to the reference sequence length, 29903. The difference between the reference and the minimum length was 1119. This does not match well with the data from the Table, according to which the maximum difference between the length reference and any other sequence is about 159 (103 mutations + 56 deletions). Moreover, with such a difference, this sequence would most likely belong to a different type of virus, since the reference sequence of SARS-CoV-2 and the bat coronavirus RaTG13 have a similar difference (GenBank MN996532.2, collection_ date=24-Jul-2013). According to (Li et al., 2023), they differ by 96.2 %, i. e. by 1136 single mutations (distributed throughout the sequence). Calculation of the distance between the same sequences, made using the Nextstrain web service, showed a difference of 1135 single mutations, as well as 20 deletions (in the coding part of RaTG13 relative to the reference sequence of SARS-CoV-2). The total genome length of RaTG13 is 28855, i. e. the number of deletions relative to SARS-CoV-2 is 48. Most probably, such too short or too long sequences correspond to low-quality data with errors in genome assembly.

**Fig. 3.**
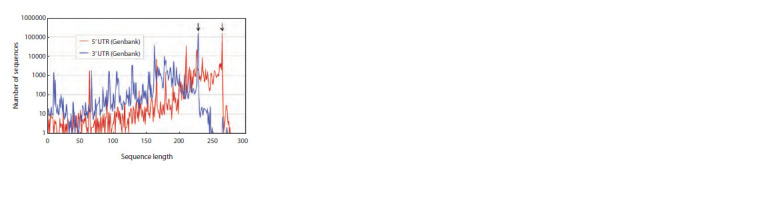
Distributions of 5’ UTR and 3’ UTR lengths for sequences from the Genbank database for the period from the emergence of SARS-CoV-2 (at the end of 2019) to the end of 2020. The lengths of 5’ UTR and 3’ UTR in the reference genome of SARS-CoV-2 are 265 and 229 nt, respectively. The peak values of both curves correspond precisely to these lengths.

**Fig. 4.**
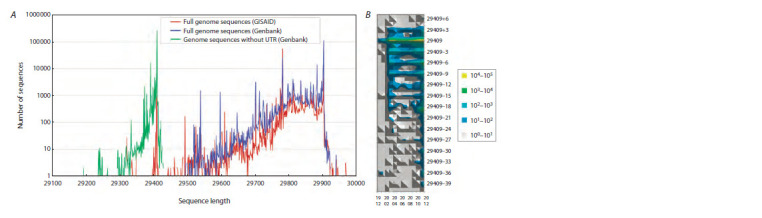
A, Distribution of lengths of full genomes (GISAID, Genbank) and their lengths without UTRs (Genbank) during a period from the emergence of SARS-CoV-2 in 2019 until the end of 2020. The full length of the SARS-CoV-2 reference genome is 29903 nt, and the length of its coding part (without UTRs) is 29409. Peak (maximal) values for all three curves correspond to these values. B, Change of the lengths of the SARS-CoV-2 genomes coding part (Genbank) during 12.2019–12.2020 by months. Horizontal signatures are numerical representations of the year and the month, vertical represent the lengths of the genome coding part; colors correspond to the frequency of genome sequences with a specified length (logarithmic scale).

Because the difference between the full length of the SARS-CoV-2 reference genome and the rest of the sequences stored in the database for some of them significantly exceeds the number of differences (point mutations, deletions and insertions) between the SARS-CoV-2 reference genome and the most different variant of “Omicron” (see the last row in the Table), we decided to study the distribution not only of

full lengths of genomes, but of their coding and non-coding regions as well (Fig. 3, 4). As seen in Figure 3, the 5′ UTR and 3′ UTR regions found in the databases have lengths from 0 to reference values, and in a small number of cases they are slightly longer. Sequences, the 5′ UTR and 3′ UTR lengths of which coincide with the reference ones, account for 49.7 and 51.2 % of their total number, respectively. Sequences, the 5′ UTR and 3′ UTR lengths of which differ from the reference ones by no more than 10 nt, constitute 55.9 and 55.7 % of their total number, respectively.

Also, Figure 4, A shows that the main source of the observed scatter in the distribution of full lengths of the SARS-CoV-2 genomes was indeed due to the scatter in lengths of the untranslated regions – 5′ and 3′ UTRs. If we consider only the coding part, the scatter is significantly reduced: 84.9 % of all sequences have the length of the coding part equal to the length of the reference genome, and 90.7 % have a length of the coding part that differs from it by no more than 10 nt. In addition, Figure 4, B shows that among the genomes, the length of the coding part of which differs from that of the reference sequence (29409), prevail those in which this difference is a multiple of 3 – to prevent a shift in the reading frame during translation, which usually leads to non-viability. Thus, most of the processed viral sequences appear to be biologically meaningful.

It can be seen that the distributions obtained based on complete genomes data from GISAID (obtained using the access through API) and Genbank (through analysis of downloaded sequences using ParSeq software) have a fairly high similarity – probably due to the fact that most sequences are stored in both databases (see Fig. 4). The question about how many sequences that differ in length from the reference one actually have deletions or insertions, and how many of them have these differences due to errors in sequencing and genome assembly, remains open.

**Fig. 5.**
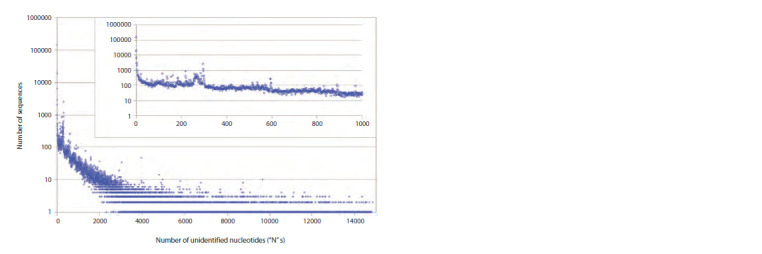
Distribution of SARS-CoV-2 sequences by the number of non-identified or partially identified nucleotides in the translatable part of their genomes (from Genbank, collection date within the interval from 12.2019 until 12.2020). The inset contains part of the same graph as in the main picture, but for the area from 0 to 1000 horizontally.

2. When studying genetic sequences representing the genomes of different variants of a virus that change over time, there is often a need to compare them with each other. Even if a pair of sequences have identical coding region lengths, the ability to calculate the amount of difference between them (the number of point mutations) will depend on whether the sequences contain undefined nucleotides, usually denoted “N”, or letters other than the standard A, T(U), G and C. Using the ParSeq software and the genomes of SARS-CoV-2 sequences collected in 2019–2020 (from the Genbank database), we calculated the distribution of sequences by the number of unidentified or partially identified nucleotides in them (Fig. 5).

Throughout most of the graph, the number of sequences decreases exponentially with the number of unidentified nucleotides, although there are areas with some peculiarities. The number of sequences for which all nucleotides are identified is 47.8 %, the number of sequences where less than 10 nucleotides are uncertain is 58.9 %. Thus, for the analysis of evolutionary changes occurring in the SARS-CoV-2 virus, a significant part of the total number of sequences is suitable.

## Discussion

We carried out a number of estimates, calculations and computational analyses (using software developed by us), to improve our understanding of the space of SARS-CoV-2 genetic sequences variants, find out what are its main properties and features associated with a quite long genomic sequence (for RNA viruses) and a low frequency of mutations occurring in the process of its replication.

There are viruses the genome of which is significantly smaller than that of SARS-CoV-2. Because of its relatively large length, the number of viable variants exceeds that of small viruses. Let’s try to determine some other landmarks in the space of viral genetic sequence variants. SARS-CoV-2 belongs to single-stranded RNA(+) viruses (Modrow et al., 2013). One on the smallest ssRNA(+) human viruses is the Astrovirus type 1 (genome length = 6771 nt) (Lewis et al., 1994). An even smaller ssRNA(+) genome (4294 nt) belongs to the shrimp nodavirus (Penaeus vannamei nodavirus) (Chen et al., 2019). The total number of variants of different sequences of these two lengths is equal to 3.533∙104076 and 1.760∙102585, correspondingly.

If in our search for the smallest viral genome we consider DNA viruses as well, then among the record holders we will find pig circovirus type 1, Porcine circovirus 1 (PCV-1) (Cao et al., 2018), with genome size equal to 1757–1759 bp (17 times less than that of SARS-CoV-2). The number of possible variants of genetic sequences of such length is 6.597∙101057. This is still a far cry from the number of variants that were potentially available to SARS-CoV-2 during the period of its existence (3.5 years), 7.985∙10511. And a genome with a length of 850 nt would have a very close number of possible sequence variants, 5.636∙10511. However, there are single-stranded circular RNA infectious agents with even shorter sequence lengths (from 246 to 467 nt), named viroids (Katsarou et al., 2015). Their RNA is not protected by any envelope and does not encode proteins.

So, SARS-CoV-2, like all other viruses, potentially has a very large number of possible variants, compared both to the number of collected and sequenced specimens, and to the number of variants that have been “tested” during evolution, but turned out to be non-viable.

And finally let’s get back to the bat coronavirus RaTG13 (L = 29855) – the nearest neighbor of SARS-CoV-2 in the space of genetic sequences variants, which differ from it by 1135 single mutations. The total number of variants of sequences generated by the reference SARS-CoV-2 genome modified by a number of mutations (from 1 to 1135), may be estimated as ≈ 2.943∙105621, which exceeds by many orders of magnitude the total number of possible variants of sequences as long as 4294 nt (1.76·102585) and 6771 nt (3.53·104076), i. e. it can contain in itself the amount of information enough for a huge number of different small viruses.

The global phylogenetic tree of the SARS-CoV-2 shows that the virus cannot remain unchanged over time; it is forced to alter, apparently due to the fact that natural selection pressure acts on it. Another reason for changes is intraspecific competition – for example, variants with faster RNA polymerases displace variants with slower ones (since their number grows faster) and thereby reduce the incubation period of the virus over time; less lethal strains allow the virus to spread longer and wider (the carrier remains alive and spreads the virus throughout almost the entire period of the disease; an infected person with mild symptoms or their absence remains socially active and infects more people in their environment). Unlike the viroids mentioned above, changes in the genome of real viruses, including SARS-CoV-2, can have different effects on intraspecific competition depending on the functions of the proteins encoded in the genome. This issue remained outside the scope of this work, but in subsequent publications we plan to pay due attention to it.

In addition, the formation of immunity to this virus in humanity also has an impact on further virus evolution, and there are probably other mechanisms too. Moreover, all these changes should occur without compromising the functionality of the virus. Thus, it turns out that the space of variants available to the SARS-CoV-2 coronavirus is quite narrow, and the trajectories of its development may be determined to some extent. Indeed, the SARS-CoV-2 genome has been shown to have a much lower mutation rate and genetic diversity compared to the SARS-CoV virus that caused the atypical pneumonia outbreak in 2002–2003 (Jia et al., 2020; Zhou et al., 2020; Nikonova et al., 2021). Thus, for example, for the SARS-CoV-2 S-protein, the dN and dS values appeared to be approximately 12 and 7 times lower than those for SARS-CoV (where dN is the fraction of sequences in a sample of genomes that contain non-synonymous mutations in a particular gene; dS is a similar value, but for synonymous mutations). For more conservative genes, ORF1a and ORF1b, the ratios of mutation frequencies

**Formula. 2.**
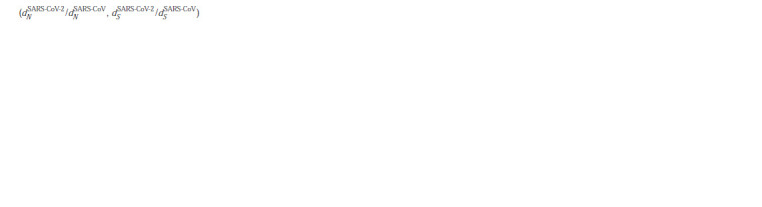
Formula 2

are less than those for S-protein, but values for SARS-CoV-2 are also lower than the corresponding values for SARS-CoV (belonging to the interval from

**Formula. 3.**
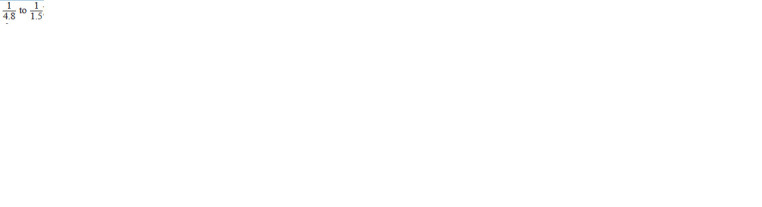
Formula 3

The hypothesis about the partial determinism of coronavirus evolutionary trajectories is that if the development of the SARS-CoV-2 pandemic, from its very beginning in December 2019, due to random factors, would have gone somewhat differently, then, despite this, sooner or later, in the same order or in a different one, the space of viable variants “visited” by the virus would still be approximately the same. The above allows to suggest that creating an evolutionary simulator based on an analysis of the trajectories of virus change over time might be quite possible, which is part of our future plans.

## Conclusion

Investigation of the space of genetic sequence variants is an important step in developing approaches for modeling the evolution of viruses and other organisms. To build a new, significantly more realistic model of virus evolution, capable of calculating potentially possible viral genome sequences variants, which are not yet realized in nature, in order to proactively prevent their emergence, it is necessary to answer questions such as: What is the probability of recombination and are there preferred positions in which it usually occurs? Can we guess or calculate which variant will be realized and which will not be viable? Could “Delta” or “Omicron” genetic sequences have been predicted (calculated before their emergence)? And finally, if it were possible to create a realistic model of the evolution of SARS-CoV-2 and calculate the process several times from the very beginning, from the initial reference sequence, would it proceed differently each time and lead to significantly different results, or would everything happen approximately the same with minor variations?

## Conflict of interest

The authors declare no conflict of interest.
